# Bacteriological Study of 117 Cases of Scarlatina Angina

**Published:** 1896-06

**Authors:** M. G. H. Lemoine


### Translation.

### BACTERIOLOGICAL STUDY OF 117 CASES OF SCARLATINA ANGINA.

By M. G. H. LEMOINE.

(Translated from *Journal de Clinique et de Therapeutique Infantiles.*)

By EVANGELINE CARROLL, M. D., Buffalo, N. Y.

THE pharyngeal accidents in scarlatina take first rank among the symptomatic manifestations of the malady by their constancy and often also by their gravity. Depending on the scarlatinal infection they seem besides to prepare a propitious soil for the development of those secondary agents, whose importance one cannot forget, in face of the facts of observation which show us in these agents the origin of the numerous complications of scarlatina.

Because of the clinical resemblance of these accidents to diphtheritic angina, a large number of observers have undertaken bacteriological researches on the subject, including these scarlatinal anginas in the works on diphtheria. These authors have revealed, however, the presence of a streptococcus, alone or combined, in the false membranes of these scarlatinal diphtherias. (Crooke, Baumgarten, Raskin, Prudden.)

Bourges and Wurtz first, in a work on scarlatinal angina, established a difference between the microbic nature of the early anginas and the late anginas of scarlatina. In eleven cases these observers found in two late anginas the bacillus of Loeffler and in nine early anginas the streptococcus, most often associated with the staphylococcus, five times. They concluded also that the early angina was due most often to the streptococcus, while the late angina was of a diphtheritic nature. M. Sevestre admitted however the possibility of the coëxistence from the outset of two infections, scarlatinal and diphtheritic.

The epidemic of scarlet fever that I observed at the beginning of the year has been for me an occasion of new researches on the subject. If these confirm in great part the observations of MM. Bourges and Wurtz, the larger number of observations and a particular technique here permitted me to acquire some new ideas, which I shall briefly expose.

These scarlatinal anginas, studied from a bacteriological point of view, number 117, plus an abscess of the tonsil supervening in a case of scarlatina on the eighteenth day of the disease.

Of the 117 anginas, 79 were pseudo-membranous and 38 showed no exudate. All belong to the period of the onset of the disease, as I have only observed a single case of late angina, which comes in elsewhere in the category of diphtheritic anginas, due to Loeffler's bacillus. Bacteriological researches have shown the presence of the streptococcus alone in 102 cases, the streptococcus associated with the bacillus of Loeffler in 5 cases, the streptococcus associated with the staphylococcus in 8 cases and the streptococcus associated with the colon bacillus in 1 case.

The results of microbiological analysis, as one may see, agree with those already obtained by MM. Bourges and Wurtz and show how constant is the presence of the streptococcus, since it is found in all the scarlatinal anginas. But, on the other hand, rare as it may be, the Loeffler bacillus may however play a part in these anginas at the outset. In fact, five times we have found this bacillus in the throat of scarlatinal patients. It is possible then, to have at the same time, as M. Sevestre has admitted, a scarlatinal and a diphtheritic infection. It is well to note that the streptococcus was obtained in a pure state in the great majority of these anginas, in 117 cases. In 102 cases it existed alone to the exclusion of other microörganisms, while in nine cases only was it associated with staphylococcus and the colon bacillus.

The explanation of the difference in our results from those previously obtained lies in the new technique which we used according to the advice of Prof. Vaillard. The technique consists essentially in the careful preparation of the material for culture, taken with care from the tonsil after the cauterisation of the surface of the organ. The implantations have always been made on agar and on serum.

If from bacteriological analysis we pass to the examination of clinical facts, we are bound to note the very particular gravity of certain cases in which the association of the strange microörganisms seem to play an important rôle. There are, above all, the bacillus of Loeffler and the colon bacillus. The other different organisms found associated with the streptococcus, either in the interior of the tonsil or in the exudate, we have not deemed it necessary to discuss, as they present such a different clinical aspect from those in which the streptococcus has been found in an isolated state. We ought also to add that the anginas, due to the streptococcus, have been very variable in their symptomatic manifestations ; some (five cases of pseudo-membranous angina) were of extreme gravity, while others have shown themselves relatively benign or of a moderate severity, attested by the well-known variability of the virulence of the streptococcus.

Four scarlatina anginas were particularly serious from the accidents by which they were accompanied during their course and during a protracted convalescence. Two were due to an association with the Loeffier bacillus and two to that with the colon bacillus. All four showed an identical clinical aspect, and the gravity of certain true diphtherias being known, in which cases the colon bacillus existed at the same time with the streptococcus, it is of great interest to note that the association gave rise to symptoms as serious as in the first two. In these, in fact, one sees unrolled the whole symptomatic picture that the old observers knew and described under the name of diphtheria—false membranes, thick, firm, adherent, leaving a bleeding surface after removal ; these membranes covering over the tonsils and uvula and presenting a grayish-white appearance. There existed at the same time sub- and retro-maxillary adenitis, painful and producing by their size the deformity of the neck, typical of patients attacked by grave diphtheria. The face pale, the fever quite high and the state of exhaustion and depression gave the impression of a profound infection of the organism. These four patients recovered, however; but one of them, who has had true diphtheritic angina, has taken five months to regain health, presenting gastric troubles and a persistent albuminuria, which disappeared only three weeks ago.

Thus, then, the association of the colon bacillus produced in two scarlatinal cases absolutely the same symptoms as the bacillus of Loeffler.

From the experiments of others, the virulence of this organism has been found to be very intense. Whereas, the streptococcus culture injected into the skin of the ear of a rabbit had only given a redness with edema and droop of the ear, accidents which were followed by recovery ; the same streptococcus injected under the same conditions, but associated with the colon bacillus of this same angina, gave instead a rapidly fatal infection (forty-eight hours).

Is it right, however, to generalise the results of these two observations ? I think not, but none the less remains the thought that the association of the streptococcus with the colon bacillus must be regarded as more dangerous than the association with the staphylococcus, the case in the last category not having given rise to any important clinical observation.

---

